# Use of endoscopic tissue morcellator in removing retroperitoneal fat in retroperitoneoscopic radical nephrectomy

**DOI:** 10.1186/s12893-020-00740-9

**Published:** 2020-04-17

**Authors:** Guang Yang, Yong Xu, Shaw P. Wan, Genyi Qu, Haibo Nie, Guangjun Duan

**Affiliations:** 1 The Affiliated Zhuzhou Hospital Xiangya Medical College CSU, ZhuZhou, China; 2 Department of Urology, The Affiliated Zhuzhou Hospital Xiangya Medical College CSU, South Changjiang Road, Tianyuan district, ZhuZhou, 412007 China; 3 The First People’s Hospital of Xiaoshan, Hangzou, China

**Keywords:** Endoscopic tissue morcellator, Radical nephrectomy, Localized renal cancer, Retroperitoneoscopic

## Abstract

**Background:**

Evaluate the safety and effectiveness of using an endoscopic tissue morcellator (ETM) to remove the retroperitoneal fat during retroperitoneoscopic radical nephrectomy (RRN).

**Methods:**

The use of ETM in the removal of retroperitoneal fat was retrospectively analyzed in patients who underwent RRN for localized renal cancer in our hospital from January 2010 to January 2018. We accrued the appropriate patients and divided them into two groups. The first group included patients of RRN where ETM was used to remove the retroperitoneal fat, while the second group was comprised of patients of RRN where ETM was not performed, which served as the control group. Each group was further divided into two subgroups, including obese patients (BMI ≥ 28) and patients suffering from high-volume renal cancer (Stage T2a). The differences between the two groups as well as their subgroups were analyzed and statistically compared.

**Results:**

All 222 nephrectomies were completed under retroperitoneoscopy, ETM was used in 105 of these 222 patients. Among them, 31 cases were of obese patients, and 26 cases were of high-volume renal cancer patients. The other 117 patients had undergone RRN without the use of ETM. Among them, 36 cases were of obese patients, and 28 cases were of high-volume renal cancer patients. The differences in age, BMI, tumor position, and tumor size between the two groups were not statistically significant, *P* > 0.05. Both the surgical time and the blood loss for the ETM group were significantly lower than the control group, *p* < 0.05. In the subgroup analysis, the obese patients and patients with high tumor volume also showed a significantly lower surgical time and less blood loss, *p* < 0.05. The postoperative hospitalization time, the total survival rate, and the disease-free survival rate were not statistically significant, *p* > 0.05.

**Conclusions:**

The use of ETM in removing the retroperitoneal fat during the RRN can potentially reduce the surgical time and lessen the blood loss. This technique is especially advantageous for obese and large-volume tumor patients.

## Background

Malignant renal tumors are generally treated by surgical excision. This can be accomplished by open, laparoscopic, or robotic-assisted radical nephrectomy. Retroperitoneoscopic radical nephrectomy (RRN) is one of the laparoscopic techniques, and it has the advantage of avoiding the intraperitoneal structures. However, one of the hindrances for the retroperitoneal approach is the retroperitoneal fat, especially in obese patients. Retroperitoneal fat is difficult to remove after it has been stripped. Often, it is left inside the retroperitoneum. In this situation, the fat can obscure the visual field and interfere with the dissection. Since 2010, in order to conduct RRN in a relatively fat-free environment, we utilized an endoscopic morcellator to remove retroperitoneal fat following its mobilization. In this study, we aimed to compare the safety and efficacy in removing retroperitoneal fat with and without ETM during RRN surgery.

## Methods

### General records

We performed a retrospective analysis of retroperitoneoscopic radical nephrectomies that were performed between January 2010 and January 2018. This project was reviewed and approved by our institutional ethics committee(No. 20100853).Initially, we accrued the appropriate patients and divided them into two groups. The first group included patients of RRN where ETM was used to remove the retroperitoneal fat, while the second group was comprised of patients of RRN where ETM was not performed, which served as the control group. Each group was further divided into two subgroups, including obese patients (BMI ≥ 28) and patients suffering from high-volume renal cancer (Stage T2a). All patients underwent an enhanced CT scan and B-mode ultrasonography before surgery. The diagnosis of renal cell carcinoma and suitability for RRN were confirmed. After surgery, Clavien-Dindo grading system was used to evaluate RRN surgical complications. All patients were followed as outpatients every 3–6 months.

### Surgery method

**Fig. 1:**
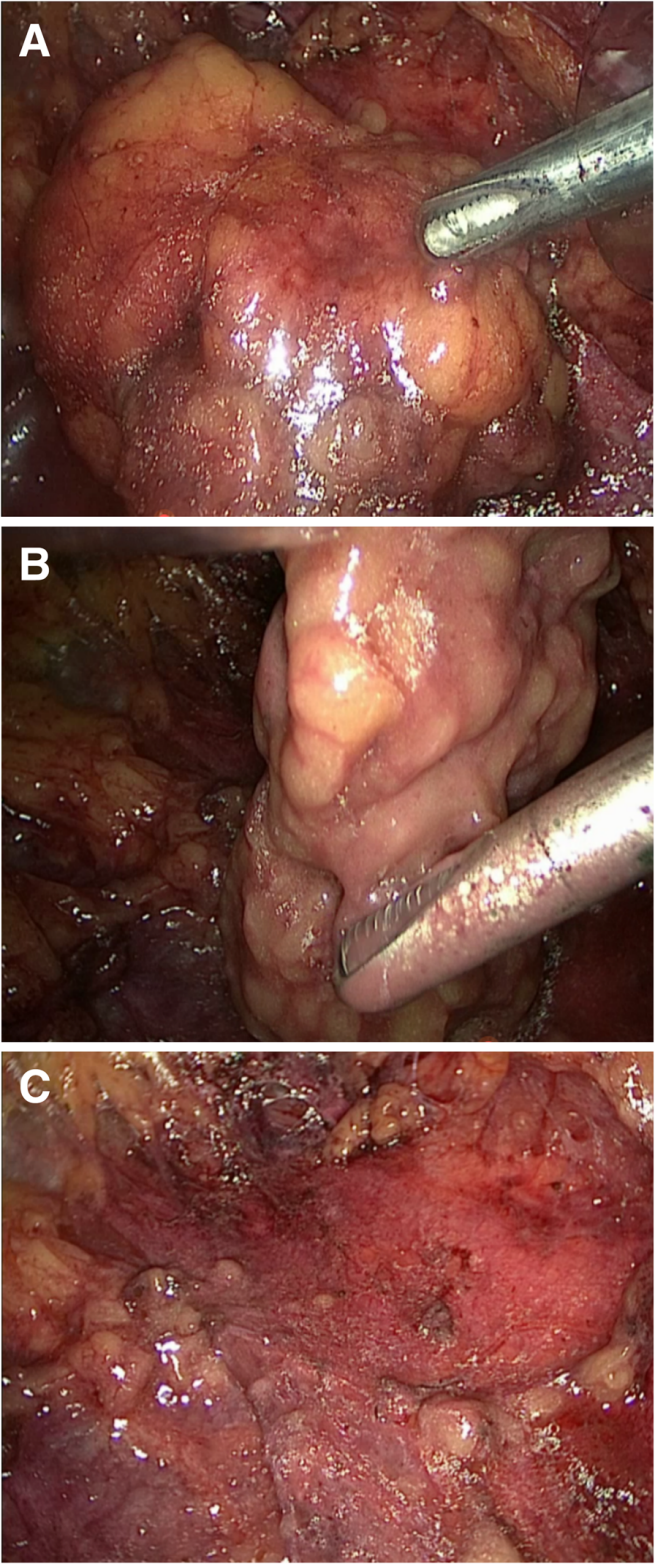
Removal of retroperitoneal fat. **a** The stripped retroperitoneal fat. **b** Use of ETM to remove retroperitoneal fat. **c** The retroperitoneum after removal of the retroperitoneal fat

Radical nephrectomy with ETM (YSB-III Endoscopic Tissue Morcellator, HAXK): The procedure was performed under general endotracheal anesthesia in supine position. Bladder was drained with an indwelling Foley catheter. A two-centimeter incision was made two centimeters above the iliac crest at the mid-axillary line. The retroperitoneal space was expanded using a balloon expander. Next, a five-millimeter trocar was inserted just below the costal margin at the posterior axillary line, and a ten-millimeter trocar was inserted just below the subcostal margin at the anterior axillary line. The trocars were inserted under the finger guide through the iliac crest incision. Upon entering the posterior abdominal cavity for positional observation, the lamellar distribution of the retroperitoneal fat was observed to be located on the surface of the anterior renal fascia and peritoneal reflex. A dissection clamp and ultrasound knife were used to strip the retroperitoneal fat, and the aforementioned retroperitoneal fat layer was removed in sheets from the diaphragmatic apex to the iliac fossa. Following its removal, the peritoneal reflex, anterior renal fascia, and their junction were visible, leaving an incised boundary between the two structures. The retroperitoneal fat was grounded and aspirated with an ETM inserted through the 10 mm trocar (Fig. 1).

Next, a longitudinal incision was made in the Gerota’s fascia near the psoas major muscle. The dissection then extended toward the diaphragm until the medial arcuate ligament on the upper surface of the psoas major was visualized. The dissection was then aimed medially to expose the renal vasculatures. The renal artery was first isolated, then secured with three Hem-o-Lok clips and divided. The renal vein was next isolated, and then similarly secured and divided. Lastly, the ureter was identified, dissected downward along the psoas major, and divided at its mid-section. The renal specimen was then completely mobilized from outside of the Gerota’s fascia and removed in its entirety. The complete specimen was removed using a specimen bag; a drain was placed through the port at the anterior axillary line; and wounds were closed.

In the control group, the nephrectomy was similarly performed but without the mobilization and removal of the retroperitoneal fat.

### Postoperative follow-up

Patient were followed regularly with serum creatinine and blood urea nitrogen, B-mode ultrasonography, CT scans, and chest X-rays at six-month intervals for the next five years and then yearly after. Patients were analyzed postoperatively by overall survival rate, disease-free survival rate, rate of death from the disease, and rate of death from unrelated causes. We also recorded the number of patients lost in the follow-up.

### Statistical processing

SPSS 22.0 statistical software was used for the analysis. Mean ± standard deviation was used to describe the measurement data. An independent sample t-test was used for the comparison of the continuous variables. An x^2^ test was used for the comparison of the categorical variables. *p* < 0.05 was considered statistically significant.

## Results

### General information

The first group included 118 cases of RRN in which ETM was used to remove the retroperitoneal fat. 13 patients were lost in follow-up and were excluded from analysis; 105 patients were included in the data analysis. Among them, 31 patients were obese as a subgroup, and 26 patients had high-volume renal cancer (Stage T2a) as another subgroup. The second group included 136 cases of RRN which were performed without the use of ETM. 19 patients were lost in follow-up and were excluded from the analysis, and the remaining 117 patients were entered into the data analysis study. Among them, 36 patients were obese as a subgroup, and 28 patients had high-volume renal cancer as another subgroup. The basic characteristics of the patients are shown in Table 1.

**Table 1:** The basic characteristics of the patients

Characteristics	the morcellator group	the control group	*p*
Number of patients	105	117	
Age(years, mean ± SD)	61.3 ± 15.9	62.8 ± 14.1	0.489
Weight (kg, mean ± SD)	68.2 ± 13.7	66.5 ± 15.8	0.094
BMI (kg/m^2^, mean ± SD)	24.6 ± 3.4	24.5 ± 2.6	0.500
Sex (n, %)			0.936
Men	58(55.2)	64(54.7)	
Women	47(44.8)	53(45.3)	
Tumor size (cm, mean ± SD)	5.6 ± 3.1	4.9 ± 3.6	0.087
Tumor side (n, %)			0.847
Right	48(45.7)	55(47.0)	
Left	57(54.3)	62(53.0)	
Clinical stage (n, %)^a^			0.886
Stage T1	79(75.2)	89(76.1)	
Stage T2a	26(24.8)	28(23.9)	
Serum creatinine (μmol/L, mean ± SD)	76.2 ± 26.7	75.2 ± 25.4	0.617

^a^ Based on the American Joint Committee on Cancer’s (AJCC) clinical staging average 151.4 ± 108.4 ml. Again, the difference significantly favored the morcellator group, *p*<0.05. The postoperative hospitalization for the morcellator group was 3–7 days, average 4.6 ± 1.2 days; the control group was 3–9 days, average 4.9 ± 1.5 days. The difference was not statistically significant, *p*>0.05. The time required to remove the retroperitoneal fat was 1.3–5.0 min, average 2.1 ± 1.8 min.

### General situation and postoperative pathology

RRN was successfully completed in all 222 patients included in the study. The differences between the age, BMI, tumor position, tumor size, and serum creatinine value in the two groups were not statistically significant, *p* > 0.05.

Surgical pathology: The morcellator group had 85 cases of clear cell renal carcinoma, 12 cases of papillary renal carcinoma, three cases of granulosa cell carcinoma, and five cases of chromophobe cell renal carcinoma. The control group had 97 cases clear cell renal carcinoma, 13 cases of papillary renal carcinoma, two cases of granulosa cell carcinoma, and five cases of chromophobe cell renal carcinoma. Surgical pathology in the two groups were found to not be statistically significant as *p* > 0.05. All decomposed fats were examined pathologically and were determined to be fat cells, however, no tumor cells were found.

### Comparison of surgery related indicators

Perioperative characteristics are presented in Table 2. The surgical time was recorded from the start of skin incision to the completion of wound closure. The surgical time for the morcellator group was 80–315 min, average 102.0 ± 42.6 min; the surgical time for the control group was 80–325 min, average 115.7 ± 53.6 min. The difference significantly favored the morcellator group, *p*<0.05. The blood loss during surgery for the morcellator group was 50–1200 ml, average 118.6 ± 105.6 ml; the control group’s blood loss during surgery was 50–1500 ml,

**Table 2:** Surgical characteristics and complications of two groups

Surgical characteristics and complications	the morcellator group (*n* = 105)	the control group (*n* = 117)	*p*
Surgical time (min, mean ± SD)	102.0 ± 42.6	115.7 ± 53.6	0.039
Mean estimated blood loss (ml, mean ± SD)	118.6 ± 105.6	151.4 ± 108.4	0.025
Hospitalization time (day, mean ± SD)	4.6 ± 1.2	4.9 ± 1.5	>0.05
Surgical complications (n, %)	8(7.6)	11(9.4)	>0.05
Peritoneum injury	6(5.7)	7(6.0)	
Wound infection	2(1.9)	4(3.4)	
Obese patients((BMI ≥ 28, n, %)	31(29.5%)	36 (30.8%)	
Surgical time (min, mean ± SD)	120.2 ± 18.8	141.5 ± 17.6	<0.001
Mean estimated blood loss (ml, mean ± SD)	127.6 ± 47.6	163.3 ± 59.2	0.007
high-volume tumors (Stage T2a, sn, %)	26 (24.8%)	28 (23.9%)	
Surgical time (min, mean ± SD)	152.7 ± 26.3	172.9 ± 17.6	0.001
Mean estimated blood loss (ml, mean ± SD)	209.6 ± 45.7	237.7 ± 61.7	<0.001

### Comparison of obese patients’ surgery related indicators

According to the standard set by the World Health Organization, a body mass index (BMI) of ≥28 is considered obese in the Chinese population. There were 31 obese patients (29.5%) in the morcellator group, and 36 obese patients in the control group (30.8%). Sub-group analysis of the surgery-related indicators in the obese patients showed that the surgery time was 110–280 min, average 120.2 ± 18.8 min, for the morcellator group; and 105–300 min, average 141.5 ± 17.6 min, for the control group. The difference significantly favored the morcellator group, *p*<0.05. The blood loss was 50–1100 ml, average 127.6 ± 47.6 ml, for the morcellator group, and 100–1200 ml, average 163.3 ± 59.2 ml, for the control group. The difference again significantly favored the morcellator group, *p*<0.05.

### Comparison of the high-volume tumor patients’ surgery-related indicators

There were 26 cases of high-volume tumors (tumor volume > 7 cm, Stage T2a) in the morcellator group (24.8%), and 28 cases in the control group (23.9%). The surgical time in the morcellator group in this subset was 130–320 min, average 152.7 ± 26.3 min. It was significantly shorter than the control group’s surgery time of 135–340 min, average 172.9 ± 17.6 min, *p*<0.05. The morcellator group’s blood loss during surgery was 150–1300 ml, average 209.6 ± 45.7 ml; again, it was significantly less than the control group’s blood loss of 200–1600 ml, average 237.7 ± 61.7 ml. The difference significantly favored the morcellator group, *p*<0.05.

### Comparison of complications and follow-up data

In the morcellator group, there were six cases of inadvertent peritoneal tears (Class II). Three were closed with titanium clips and three were managed with peritoneal drainage. There were two cases of minor postoperative wound infections (Class I). The median postoperative follow-up time was 49 (3–91) months. Seven patients died of unrelated causes. 13 patients were lost in the follow-up, and were excluded from the statistical analysis. Two patients developed distant metastasis, both Stage T2a patients. No patients developed local recurrence. The overall survival rate was 83.1%.

In the control group, there were seven cases of peritoneal tears (Class II). Three were closed with titanium clips and four were managed with peritoneal drainage. There were four cases of minor postoperative wound infections (Class I). The median postoperative follow-up time was 47(3–93) months. Five patients died of unrelated causes. 19 patients were lost in follow-up, and were excluded from the statistical analysis. Two patients developed distant metastasis, both Stage T2a patients. No patients developed local recurrence. The overall survival rate was 82.3%.

## Discussion

Laparoscopic radical nephrectomy has become a common surgical technique for the treatment of renal tumors that are not suitable for nephron-sparing surgery. Clayman et al. [1] first reported on transperitoneal laparoscopic radical nephrectomy. Soon afterward, Gaur et al. [2] reported on retroperitoneal radical nephrectomy.

The advantages of the transperitoneal approach are the following: the approach provides a relatively large operative field; the anatomical landmarks are easier to identify than the retroperitoneal route; and the anterior approach makes it easier to expose and secure the renal hilum. However, the approach involves the risk of inadvertent injury to the intestines and other intra-abdominal organs. There is also the potential of postoperative intra-abdominal adhesions [3, 4]. A retroperitoneal approach can avoid the intra-abdominal organs and lessen the risk of postoperative adhesions [5]. However, this approach has a relatively small operative field and the anatomical landmarks are harder to identify. The retroperitoneal fat, especially in the obese patients, can lessen these disadvantages [4]. ETM is intended for use under direct or endoscopic visualization for the morcellation and removal of dissected tissue [6]. We found that ETM can be used to remove retroperitoneal fat in order to increase the operative space and improve the visual field. The device is quite simple and safe to operate in the retroperitoneal space. The time required to remove the retroperitoneal fat averaged two minutes.

Employing ETM reduced the surgical time and mitigated the amount of blood loss, which were more pronounced in obese patients and those with large-volume tumors as obese patients possess more retroperitoneal lipids, resulting in additional interference during RRN. Retroperitoneal laparoscopic radical nephrectomy takes tumor size and stage limitations into account. If a renal tumor’s volume is greater than 7 cm, the difficulty in performing RRN increases. We believe that large-volume tumors mainly occupy the majority of the retroperitoneal space, creating difficulties for the surgeon. However, adopting ETM in the management will completely eliminate the occurrence of retroperitoneal lipids, providing more space during surgery as well as a wider viewing angle, thus reducing surgical difficulty. The use of ETM has no impact on the overall survival rate or the disease-free survival rate and did not affect hospitalization time. One important factor that should be mentioned is that the ETM should not be used for the perinephric fat that is inside the Gerota’s fascia. In this case, the tissue should be removed en bloc with the kidney.

The major weakness of this study is that it is a retrospective study. A prospective randomized study would be more conclusive. In this study, patients with stage T1 and T2a renal cancer were mainly evaluated, but the safety and efficacy of using ETM in RRN for patients in their middle and advanced stages were not further evaluated. However, we believe our data clearly showed the advantages of using ETM in retroperitoneal radical nephrectomy.

## Conclusion

The use of tissue morcellator in removing the retroperitoneal fat during retroperitoneoscopic radical nephrectomy can reduce surgical time and lessen blood loss. These advantages are more pronounced in obese patients and in patients who have large-volume renal tumors.

## Data Availability

The datasets used and/or analysed in the current study are available from the corresponding author upon reasonable request.

## Abbreviations

ETM: Endoscopic tissue morcellator
RRN: Retroperitoneoscopic radical nephrectomy
